# A new phenothiazine derivate is active against *Clostridioides difficile* and shows low cytotoxicity

**DOI:** 10.1371/journal.pone.0258207

**Published:** 2021-10-01

**Authors:** Troels Ronco, Francisca Maria Aragao, Lasse Saaby, Jørn B. Christensen, Anders Permin, Andrew R. Williams, Stig M. Thamsborg, Rikke H. Olsen

**Affiliations:** 1 Department of Veterinary and Animal Sciences, Faculty of Health and Medical Sciences, University of Copenhagen, Copenhagen, Denmark; 2 Department of Chemistry, Faculty of Science, University of Copenhagen, Copenhagen, Denmark; 3 Unibrains, Virum, Denmark; University of Illinois at Chicago, UNITED STATES

## Abstract

The rapid evolution of antibiotic resistance in *Clostridioides difficile* and the consequent effects on prevention and treatment of *C*. *difficile* infections (CDIs) are matters of concern for public health. Thioridazine, a compound belonging to the phenothiazine group, has previous shown antimicrobial activity against *C*. *difficile*. The purpose of this present study was to investigate the potential of a novel phenothiazine derivative, JBC 1847, as an oral antimicrobial for treatment of intestinal pathogens and CDIs. The minimal inhibition concentration and the minimum bactericidal concentration of JBC 1847 against *C*. *difficile* ATCC 43255 were determined 4 μg/mL and high tolerance after oral administration in mice was observed (up to 100 mg/kg bodyweight). Pharmacokinetic modeling was conducted *in silico* using GastroPlus^TM^, predicting low (< 10%) systemic uptake after oral exposure and corresponding low C_max_ in plasma. Impact on the intestinal bacterial composition after four days of treatment was determined by 16s rRNA MiSeq sequencing and revealed only minor impact on the microbiota in non-clinically affected mice, and there was no difference between colony-forming unit (CFU)/gram fecal material between JBC 1847 and placebo treated mice. The cytotoxicity of the compound was assessed in Caco-2 cell-line assays, in which indication of toxicity was not observed in concentrations up to seven times the minimal bactericidal concentration. In conclusion, the novel phenothiazine derivative demonstrated high antimicrobial activity against *C*. *difficile*, had low predicted gastrointestinal absorption, low intestinal (*in vitro*) cytotoxicity, and only induced minor changes of the healthy microbiota, altogether supporting that JBC 1847 could represent a novel antimicrobial candidate. The clinical importance hereof calls for future experimental studies in CDI models.

## Introduction

*Clostridium difficile*, recently reclassified as *Clostridioides difficile*, is a Gram-positive anaerobic pathogen recognized as the major cause of healthcare antibiotic-associated diarrhea [1]. Antibiotics used for treating every kind of infection may potentially promote *C*. *difficile* infection (CDI) [2, 3]. After antibiotic therapy, the protective intestinal microbiota is disrupted allowing ingested or resident *C*. *difficile* to (re)colonize the gastrointestinal tract and infect the host. Antibiotic resistance enables *C*. *difficile* to grow in the presence of drugs, so strains resistant to multiple agents may have a selective advantage for their diffusion from the usage of these antibiotics [4, 5]. Worldwide, CDIs constitute a remarkable public health issue and previously, *C*. *difficile* has been reported the most common health care–associated pathogen in the United States [3, 6]. Concordantly, CDC has announced *C*. *difficile* as a major pathogen [7] and is currently on a list of the five bacterial pathogens for which novel antimicrobial are urgently needed (https://www.cdc.gov/cdiff/index.html).

Phenothiazines, a group of medical compounds intended for the treatment of mental disorders, has been shown to also possess antimicrobial activity against *C*. *difficile* and various others Gram-positive pathogens [8, 9]. We have previously synthesized a phenothiazine (promazine) derivative, JBC 1847, which has been shown to lower the minimal inhibition concentration (MIC) remarkably against Methicillin resistant *Staphylococcus aureus* (MRSA) compared to the mother compound, promazine [10]. Furthermore, the novel compound does not cross the blood-brain-barrier (BBB) [10] in contrast to original phenothiazines which are rapidly available systemically after oral administration [11] and readily pass the BBB [12]. The aim of the present study was to determine the antimicrobial activity of JBC 1847 against *C*. *difficile* and to assess the intestinal cytotoxicity, oral tolerance and *in vivo* impact on the intestinal microbiota (species composition and bacterial count) of the compound. Altogether, the investigations will provide insight of the potential of JBC 1847 as a candidate for future oral antimicrobial therapy against intestinal pathogens.

## Materials and methods

### Compound characteristics and bacterial strains

JBC 1847 was synthesized at University of Copenhagen, Denmark, as previously described and initial investigations on the antimicrobial and physicochemical properties of JBC 1847 reported [10]. In this study, an advanced compartment and transit (ACAT) model using GastroPlus^TM^ software (Simulations Plus, Inc., Lancaster, CA) was used. This model incorporates physicochemical (i.e., solubility and permeability), physiological (i.e., regional pH and transit time along the gastrointestinal tract), and pharmacokinetic (i.e., clearance and volume of distribution) parameters and other factors (such as dose and P-glycoprotein [Pgp] substrate data) to predict exposure. The input was set to 100 mg JBC 1847 in tablet form administrated to a 70 kg human individual.

*C*. *difficile* ATCC 43255, previously isolated from an abdominal wound, was used to assess the anti-clostridioides activity of JBC 1847. The strain was stored in reinforced clostridial medium (Oxoid, Roskilde, Denmark) in 15% glycerol at -80⁰C until use and grown anaerobically on ChromID® *C*. *difficile* plates (bioMérieux, Ballerup, Denmark).

### Ethics statement

All animal procedures were carried out at Statens Serum Institut (SSI), Copenhagen, Denmark, and approved under a Danish Animal Experiment Expectorate. SSI has an Animal Welfare Committee which is equivalent to IACUC (American Association for Laboratory Animal Science) and provides general guidelines for all animal experiments conducted at SSI. Therefore, we declare that all ethical principles and guidelines for experiments on animals where carefully followed.

### Determination of growth inhibitory and bactericidal concentrations

The MIC of JBC 1847 was determined in 96-well microtitre plates using bacterial inocula of ∼ 10^6^ Colony Forming Units (CFU)/mL of *C*. *difficile* 43255 and were defined as the lowest concentrations of JBC 1847 compounds that inhibited visible growth after 24 h of anaerobic incubation. Minimum bactericidal concentration (MIC) values were defined as the lowest concentrations of test compounds causing ≥3 log reductions of the initial cell inocula (∼ 10^5^ CFU/mL in the final assay). Briefly, 100 μl from each well of the MIC assay (after incubation) were plates on ChromID® plates. After incubation for 24 h, viable counts were enumerated on ChromID® plates. Both the MIC and MBC were conducted with three biological replicates.

### Caco-2 cell-line cytotoxicity of JBC 1847

Caco-2 cells were seeded onto to permeable supports (T12, Corning cci-3401) and cultured for 21 days. Culture medium was changed every other day in both the apical and basolateral chamber. On the day of transport, the cells were equilibrated to ambient temperatures and the transepithelial electrical resistance (TEER) was measured across the cell monolayers. Subsequently, the cell layers were washed twice with supplemented HBSS (0.05% BSA, 10 mM HEPES) and the TEER was measured again. The exposure experiment was started by replacing the supplemented HBSS in the apical chamber with HBSS solutions of different concentrations of JBC1847.3 (0, 1, 5, 10, 25, 50 and 100 μM). After 2 h exposure samples for lactate dehydrogenase (LDH) assay were taken and the cells were washed twice in supplemented HBSS and TEER measured.

The cell proliferation assay (MTS/PMS; [3-(4,5-dimethylthiazol-2-yl)-5-(3-carboxymethoxyphenyl)-2-(4-sulfophenyl)-2H-tetrazolium, inner salt; MTS] and an electron coupling reagent (phenazine methosulfate) PMS) assay was performed by incubating the Caco-2 cell monolayers with 400 μL of a PMS/MTS solution in the apical compartment and 500 μL supplemented HBSS in the basolateral chamber (according to manufacturer’s instructions) for 60 minutes at 37°C and circular rotation (50 rpm). After incubation, 2×100 μL samples were taken from each monolayer and the absorbance at 492 nm in a 96-well plate.

For analysis of the LDH activity, the samples taken from the apical compartment after the exposure experiment were diluted 5-fold and analyzed in the LDH assay according to manufacturer’s instructions. Briefly, the assay consisted of mixing the diluted samples with different assay reagents followed by incubation at room temperature for 30 min and the absorbance at 492 nm as a measure for LDH activity. A lysate of Caco-2 monolayers (prepared by suspending Caco-2 cell monolayers in purified water) was included as a positive control for maximal response in the assay.

### Multiple toxicity dose in mice

After completion of initial *in vitro* and toxicity screening, investigations on the maximal tolerable dose (MTD) of JBC 1847 was conducted. The MTD was conducted with escalating doses from 5 to 100 mg/kg bodyweight (bw). The mice were scored for clinical signs of discomfort for 4 h. The mice were intensively monitored, and scored according to the following parameters: “0” if there were no signs of clinical discomfort; “1” if there were minor clinical signs of discomfort e.g. slower movements, light piloerection in the skin, light tucked up belly; “2” if there were moderate signs of discomfort e.g. lack of curiosity, movements only when pushed, piloerection in the skin, tucked up belly, “3” Clear signs of discomfort e.g. no movements, piloerection in the skin, tucked up belly, pinched eyes. When moderate discomfort was observed mice were euthanized and the dose considered not tolerated. Mice with no clinical scores within 4 h after peroral dosing were dosed once more with the same dose. Mice were scored for an additional 2 h after the last dose. Briefly, approx. ½ h prior to dosing, NMRI female mice, 26–30 gram (Taconic), were treated orally with 45 μl nurofen (20 mg ibuprofen/ml corresponding to approx. 30 mg/kg) as a pain relief. The dosing was based on a mean weight of 28 g per mouse. Two mice were used per dosing group, starting with the lowest dose, and mice were observed closely for a minimum of 10 min before proceeding to a higher dose.

### Impact of JBC 1847 on microbiome composition and bacterial counts in fecal samples

The MTD studies (described above) revealed that the mice tolerated JBC 1847 in concentrations up to 100 mg/kg bw without any clinical signs of discomfort. In addition, two doses of 100 mg/kg (administered with 4 h interval) were also tolerated well by all treated mice. To investigate if there was an impact of JBC 1847 on the gastrointestinal microbiota in the non-clinical mice, and if the possible impact was concentration-dependent, a second murine study was conducted. Three treatment groups (vehicle, 20 mg/kg and 100 mg/kg, respectively) each consisting of five mice (C57BL/6, Female 5 weeks old, Taconic) were treated once daily for 4 days. Mice were treated by oral gavage with 0.2 ml JBC 1847, in final dose level of 20 mg/kg bw or 100 mg/kg bw or with vehicle twice daily. Mice were weighed once daily and observed for clinical signs of discomfort and euthanized if moderately affected (score 2). Feces were collected before treatment was initiated, directly from each mouse individually. The bedding in the cages were changed once daily after treatment. On day 5 (one day after ended treatment) feces were again collected and mice were euthanized, and the entire small intestine from each collected and rinsed from the content. The epithelial layer was scraped off from the entire small intestine of each sample with a sterile microscope slide and collected in a sterile container. Both fecal and intestinal samples were stored at -20⁰C before DNA extraction.

Sample DNA extraction was done using a slightly modified version of the standard protocol for FastDNA Spin kit for Soil (MP Biomedicals, USA) with the following exceptions. 500 μL of sample, 480 μL Sodium Phosphate Buffer and 120 μL MT Buffer were added to a Lysing Matrix E tube. Bead beating was done at 6 m/s for 4 ×40 s [13]. Gel electrophoresis using Tapestation 2200 and Genomic DNA screentapes (Agilent, USA) was used to validate product size and purity of a subset of DNA extracts. DNA concentration was measured using Qubit dsDNA HS/BR Assay kit (Thermo Fisher Scientific, USA).

For the sequencing library preparation, the archaea and Bacteria, 16S rRNA gene region V4 sequencing libraries were prepared by a custom protocol based on an Illumina protocol (Illumina, 2015). Up to 10 ng of extracted DNA was used as template for PCR amplification of the Archaea and Bacteria, 16S rRNA gene region V4 amplicons. Each PCR reaction (25 μL) contained (12.5 μL) PCRBIO Ultra mix and 400 nM of each forward and reverse tailed primer mix. PCR was done with the following program: Initial denaturation at 95⁰C for 2 min, 30 cycles of amplification (95⁰C for 15 s, 55⁰C for 15 s, 72⁰C for 50 s) and a final elongation at 72⁰C for 5 min. Duplicate PCR reactions were performed for each sample and the duplicates were pooled after PCR. The forward and reverse, tailed primers were designed according to (Illumina, 2015) and contain primers targeting the Archaea and Bacteria, 16S rRNA gene region V4: [515FB] TGYCAGCMGCCGCGGTAA and [806RB] GGACTACNVGGGTWTCTAAT [14] The primer tails enable attachment of Illumina Nextera adaptors necessary for sequencing in a subsequent PCR. The resulting amplicon libraries were purified using the standard protocol for CleanPCR SPRI beads (CleanNA, NL) with a bead to sample ratio of 4:5. DNA was eluted in 25 μL of nuclease free water (Qiagen, Germany). DNA concentration was measured using Qubit dsDNA HS Assay kit (Thermo Fisher Scientific, USA). Gel electrophoresis using Tapestation 2200 and D1000/High sensitivity D1000 screentapes (Agilent, USA) was used to validate product size and purity of a subset of sequencing libraries. Sequencing libraries were prepared from the purified amplicon libraries using a second PCR. Each PCR reaction (25 μL) contained PCRBIO HiFi buffer (1×), PCRBIO HiFi Polymerase (1 U/reaction) (PCRBiosystems, UK), adaptor mix (400 nM of each forward and reverse) and up to 10 ng of amplicon library template. PCR was done with the following program: Initial denaturation at 95⁰C for 2 min, 8 cycles of amplification (95⁰C for 20 s, 55⁰C for 30 s, 72⁰C for 60 s) and a final elongation at 72⁰C for 5 min. The resulting sequencing libraries were purified using the standard protocol for CleanPCR SPRI beads with a bead to sample ratio of 4:5. DNA was eluted in 25 μL of nuclease free water. DNA concentration was measured using Qubit dsDNA HS Assay kit. Gel electrophoresis using Tapestation 2200 and D1000/High sensitivity D1000 screentapes was used to validate product size and purity of a subset of sequencing libraries. The purified sequencing libraries were pooled in equimolar concentrations and diluted to 2 nM. The samples were paired-end sequenced (2 ×x300 bp) on a MiSeq (Illumina, USA) using a MiSeq Reagent kit v3 (Illumina, USA) following the standard guidelines for preparing and loading samples on the MiSeq. > 10% PhiX control library was spiked in to overcome low complexity issues often observed with amplicon samples. In the Bioinformatic processing the forward and reverse reads were trimmed for quality using Trimmomatic v. 0.32 [15] with the settings SLIDINGWINDOW:5:3 and MINLEN: 225. The trimmed forward and reverse reads were merged using FLASH v. 1.2.7 [16] with the settings -m 10 -M 250. The trimmed reads were dereplicated and formatted for use in the UPARSE workflow (Edgar, 2013). The dereplicated reads were clustered, using the usearch v. 7.0.1090 -cluster_otus command with default settings. OTU abundances were estimated using the usearch v. 7.0.1090 -usearch_global command with -id 0.97 -maxaccepts 0 -maxrejects 0. Taxonomy was assigned using the RDP classifier [17] as implemented in the parallel_assign_taxonomy_rdp.py script in QIIME [18] using–confidence 0.8 and the SILVA database, release 132 [19]. All bioinformatic processing was done via RStudio IDE (1.2.1335) running R version 4.0.2 (2020-06-22) and using the R packages: ampvis (2.7.0) [13], tidyverse (1.3.0), seqinr (4.2.5), ShortRead (1.46.0) and iNEXT (2.0.20) [20]. For quantification of CFU per gram feces, the abundance of 16S rRNA genes per ng of isolated DNA was estimated based on the broad-range qPCR probe and primer set [21]. Briefly, the forward (5’-TCCTACGGGAGGCAGCAGT-3’) and reverse (5’GACTACCAGGGTATCTAATCCTGTT-3’) primers were used to amplify the qPCR amplicon of from *E*. *coli* MG1655 using the AccuPrime Pfx DNA polymerase (Thermo Scientific). The PCR was carried out according to the manufacturers recommendations with the following PCR program: PCR activation (94⁰C, 2 min) followed by 30 cycles of denaturation (94⁰C, 30 s), annealing (60⁰C, 30 s) and extension (68⁰C, 90 s) and a final extension (68⁰C, 5 min). The PCR product was purified on a E-gel Clone ell gel (Thermo Scientific) and cloned into the pCR4-TOPO vector using the TOPO TA CloningKit for Sequencing (Thermo Scientific) according to the manufactures recommendations. The obtained plasmid subsequently linearized with FastDigest NcoI (Fermentas), blunted using the Klenow fragment (Fermentas). The concentration for the amplicon stock was determined using the Qubit HS dsDNA assaykit (Life Technologies) and the copy number then calculated based on the molecular weight of the linearized plasmid. The amplicon stock was diluted to 108 copies/μL in 10 mM tris buffer (pH 8.5) and stored as aliquots at -18°C. Sample qPCR measurements were done in technical duplicates using the Mx3005P qPCR system (Stratagene) and the EXPRESS qPCR Supermix (Life Technologies). Reactions of 24 μL were prepared according to manufacturer’s instruction using 50 nM ROX, 500 nM of each primer, 200 nM hydrolysis probe (6-FAM)-5’-CGTATTACCGCGGCTGCTGGCAC- 3’-(BHQ-1) and 1 μL template DNA. The qPCR reaction conditions were as follows: UDG incubation (50°C, 2 min) and PCR activation (95°C, 2 min) followed by 45 cycles of denaturation (95°C, 15 s) and combined annealing and extension (60°C, 1 min). Amplicon standards with concentrations ranging from 103–107 copies/μL were included for all qPCR runs and used for quantification. A clear logarithmic correlation was found between amplicon concentration and the Cq value (R2 > 0.99) and the efficiency of the qPCR was acceptable (> 90%). All primers and probes were HPLC purified (DNA Technology, Denmark).

## Results

### JBC 1847 physiochemical and pharmacokinetic properties

Computer simulated (in silico) physiochemical and pharmacokinetic properties of 100 mg of JBC 1847 was predicted in GastroPlus^TM^.

The total overall absorption of JBC 1847 was predicted to be 6.4%, and the regional absorption through different segments (stomach, duodenum, jejunum-1, jejunum-2, ileum-1, ileum-2, ileum-3, caecum and ascending colon) differed from 0–1.5%. The models predicted the plasma concentration–time profiles of JBC 1847 with C_max_ 0.046 μg/mL, T_max_ 4.32 h and AUC0-t (μg/mL-h/mL) (Table 1)

**Table 1:** In silico predictions of physiochemical and pharmacokinetic properties of JBC 1847.

Parameter	Value
Molecular weight	423
pKa	2.5
Mean partical density (g/ml)	1.2
Diffusion coefficient in aqueous med (cm^2/sec^)	0.56
Blood/plasma concentration ration	0.78
Adjusted unbound fraction in plasma (%)	7.26
Renal clearance T_1/2_ (h)	1.83
Central compartment volume (L/kg)	0.6
C_max_ (μg/mL)	0.046
T_max_ (h)	4.32
AUC 0-t (μg/mL-h/mL)	0.33
C_max_ liver (μg/mL)	0.06
Total absorbed (%)	6.4
Stomach	0
Duodenum	0.1
Jejunum -first part/second part	1.4/1.5/
Ileum–first, middle, last part	1.3/0.9/0.6/
Caecum	0.3
Ascending colon	0.3
Small intestine transition time (h)	3.2

The table shows pharmacological parameters predicted in the GastroPlus™, version 9.6 Model. The input data is: Oral administration of 100 mg JBC 1847 (tablet); 70 kg fasted human. Molecular weight, pKa and Mean partical density are given parameters.

### Antimicrobial activity on *C*. *difficile* viable cells

MIC of JBC 1847 was determined to be 4 μg/mL. Culturing of *C*. *difficile* exposed to this concentration did not reveal any viable bacteria; hence, the MBC equaled the MIC value of 4 μg/mL.

### Cytotoxicity

**Fig 1:**
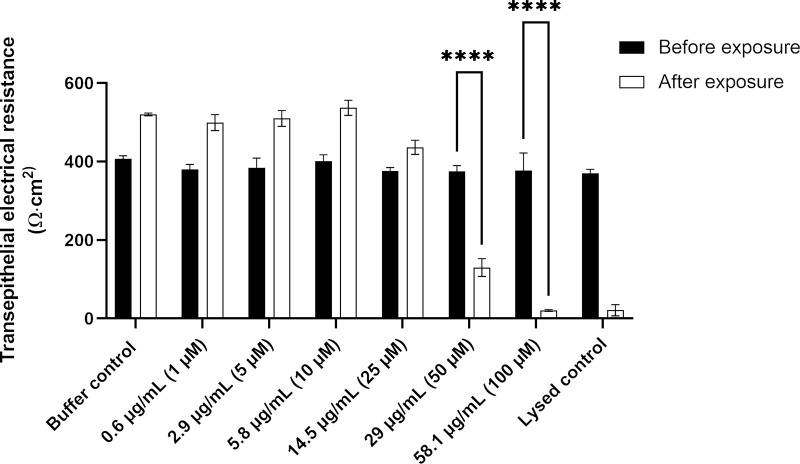
Transepithelial electrical resistance of JBC 1847. The figure shows the transepithelial electrical resistance (TEER) measured across Caco-2 cell monolayers before and after exposure to different concentrations of JBC 1847. Values on the x-axis represent measured TEER values across cell monolayers exposed to either transport buffer, different concentrations of JBC 1847 (0.6 μg/ml- 58.1 μg/ml) or purified water. Data is shown as mean ± SD of triplicates (n = 3). The measured TEER after exposure to 29 μg/ml and concentrations above was significantly reduced (*) compared to the measured resistance before exposure.

The TEER of Caco-2 cell monolayers exposed to different concentrations of JBC 1847 revealed no reduction in TEER-values in the monolayers exposed to JBC1847 in concentrations up to 14.5 μg/mL (25 μM) compared to the buffer (negative) control (Fig 1).

In contrast, the measured TEER values across the monolayers after 2 h exposure to 29 μg/mL (50 μM) and 58.1 μg/mL (100 μM) were significantly reduced compared to TEER values before exposure (ordinary 2-way ANOVA, α = 0.05). The TEER-value measured after exposure to 58.1 μg /mL was at level with the lysed control samples, which indicates that the barrier properties for these Caco-2 cell monolayers were completely compromised.

**Fig 2:**
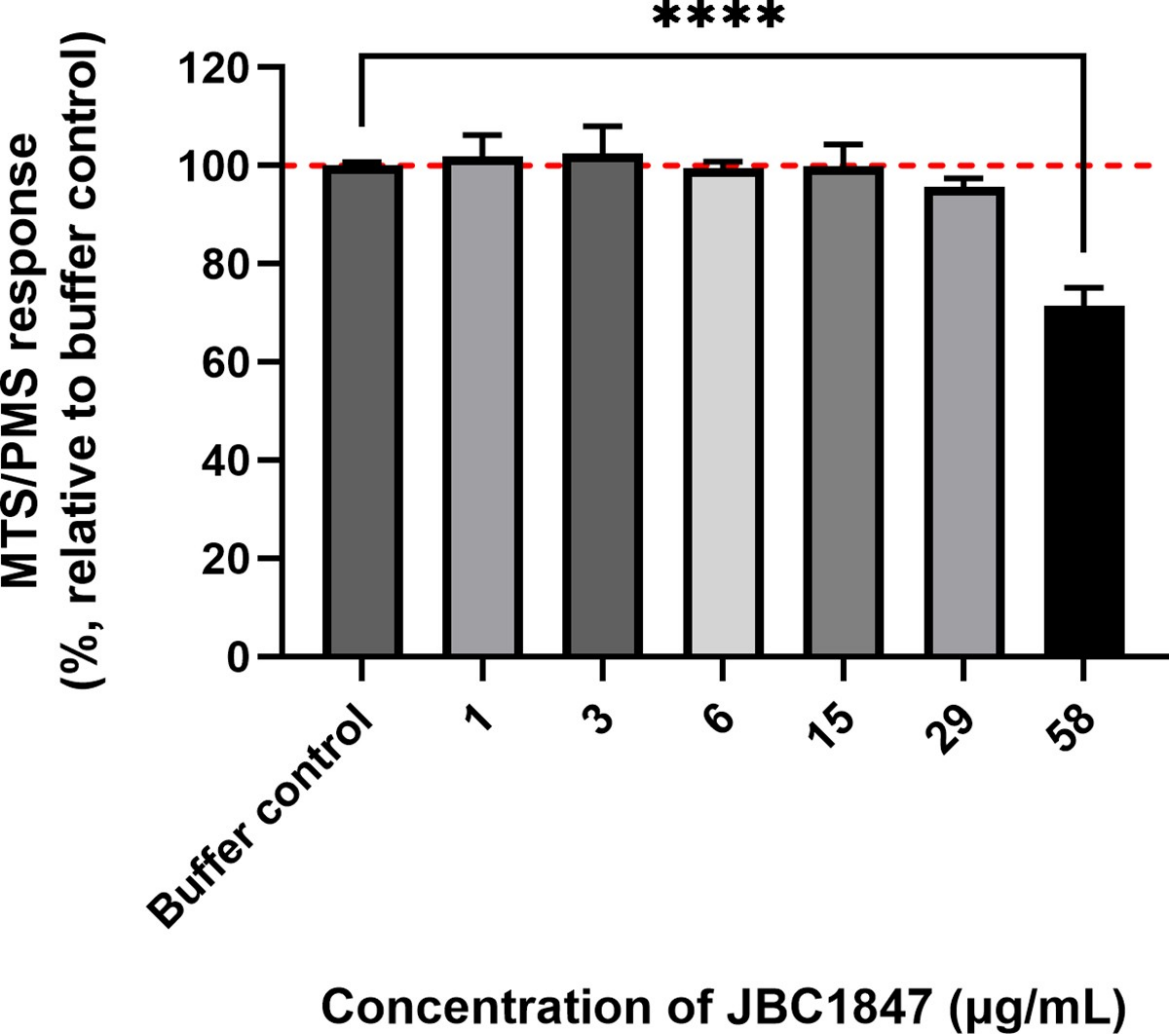
MTS/PMS assay. The figure shows the results of the MTS/PMS assay performed on Caco-2 cell monolayers exposed to either transport buffer or different concentrations of JBC 1847 (0.6 μg/ml- 58.1 μg/ml). Data is shown as assay response (absorbance value at 490 nm) of the different samples relative to the mean response measured for the buffer control samples (mean ±SD). All samples were run in triplicate. The horizontal dashed line indicates the mean response level for buffer control samples (100%). Only the response.

From the MTS/PMS assay, which is a measure of metabolic activity in the cells, it was evident that only Caco-2 cell monolayers exposed to 58.1 μg/mL of JBC 1847 exhibited a statistically significant reduction in MTS/PMS response (ordinary one-way ANOVA, α = 0.05) (Fig 2).

This result somewhat reflects the TEER data shown in Fig 1, but with the exception that the response from Caco-2 cell monolayers exposed to 29 μg/mL JBC 1847 was not significantly reduced compared to buffer controls. However, from Fig 2, it can be seen that the response from cell monolayers exposed to 29 μg/mL JBC 1847 appears to be slightly below the dashed line marking the response from buffer control samples.

**Fig 3:**
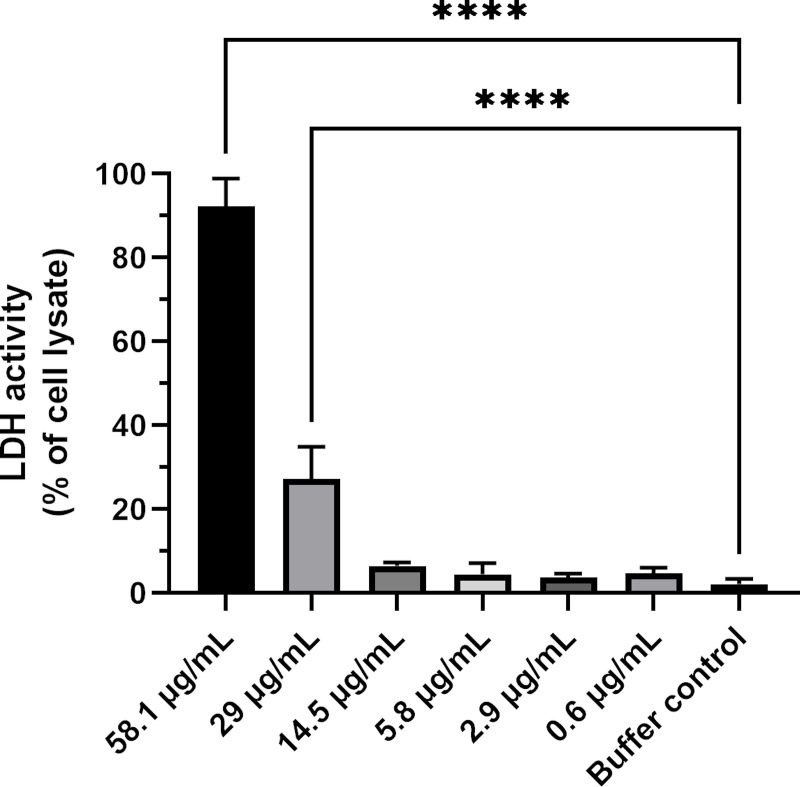
LDH assay. The LDH assay was performed on samples taken from the apical side of Caco-2 cell monolayers exposed to either transport buffer or different concentrations of JBC 1847 (0.6 μg/ml- 58.1 μg/ml) or purified water. Data is shown as assay response (absorbance value at 492 nm) of the different samples relative to the mean response measured for samples from the Caco-2 cell monolayer exposed to purified water (mean±SD). All samples were run in triplicate. Responses measured from Caco-2 cell monolayers exposed to 58.1 μg/ml and 29 μg/ml were significantly higher in buffer control in an ordinary one-way ANOVA.

The results of the LDH assay of samples taken from the apical side of Caco-2 cell monolayers exposed to either supplemented HBSS buffer (buffer control), different concentrations of JBC1 8473 or purified water (cell lysis control, 100% release of LDH), showed that samples from cells exposed to 0.0581 μg /mL (100 μM) and 29 μg/mL (50 μM) had a statistically significant increase in LDH release compared to the buffer control samples (ordinary one-way ANOVA, α = 0.05) (Fig 3).

### Oral administration and impact on the microbiota

In the MTD, the mice were intensively monitored, and scored according to the following parameters outlined in the methods sectionOne mouse out of 10 ice scored “1” after the second dosage of 25 mg/kg. All remaining mice scored “0”, even at the highest used dose (2 ×100 mg/kg bw administered at four h interval).

**Fig 4:**
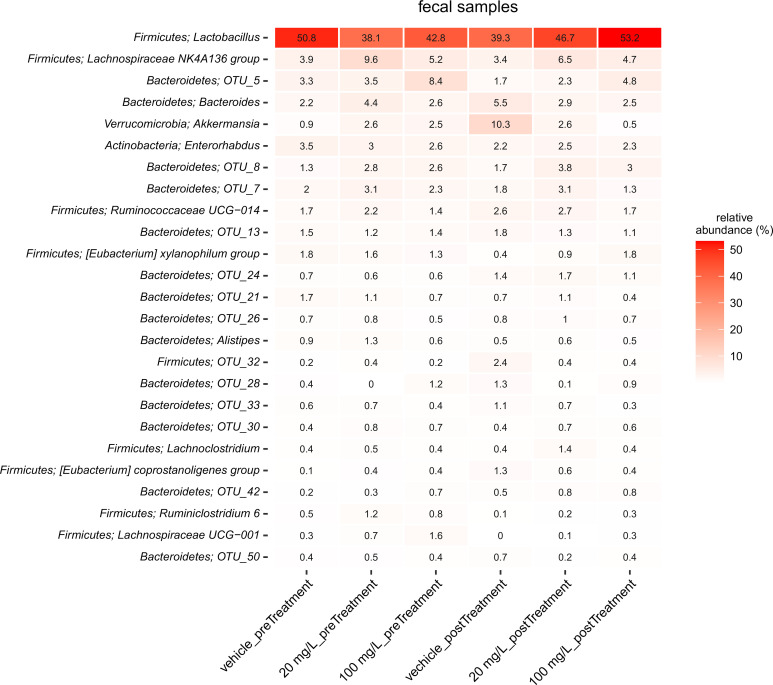
Microbial composition in fecal samples. The figure shows a heatmap of the 25 most abundant genera in fecal samples obtained before treatment was initiated (preTreatment) and the day following four days of treatment (postTreatment). Where available the OTU’s phylum classification is provided along with genus, and if no genus level classification could be obtained, the lowest assigned taxonomic classification is given. Values are shown as normalized fraction of total sequences (%).

**Fig 5:**
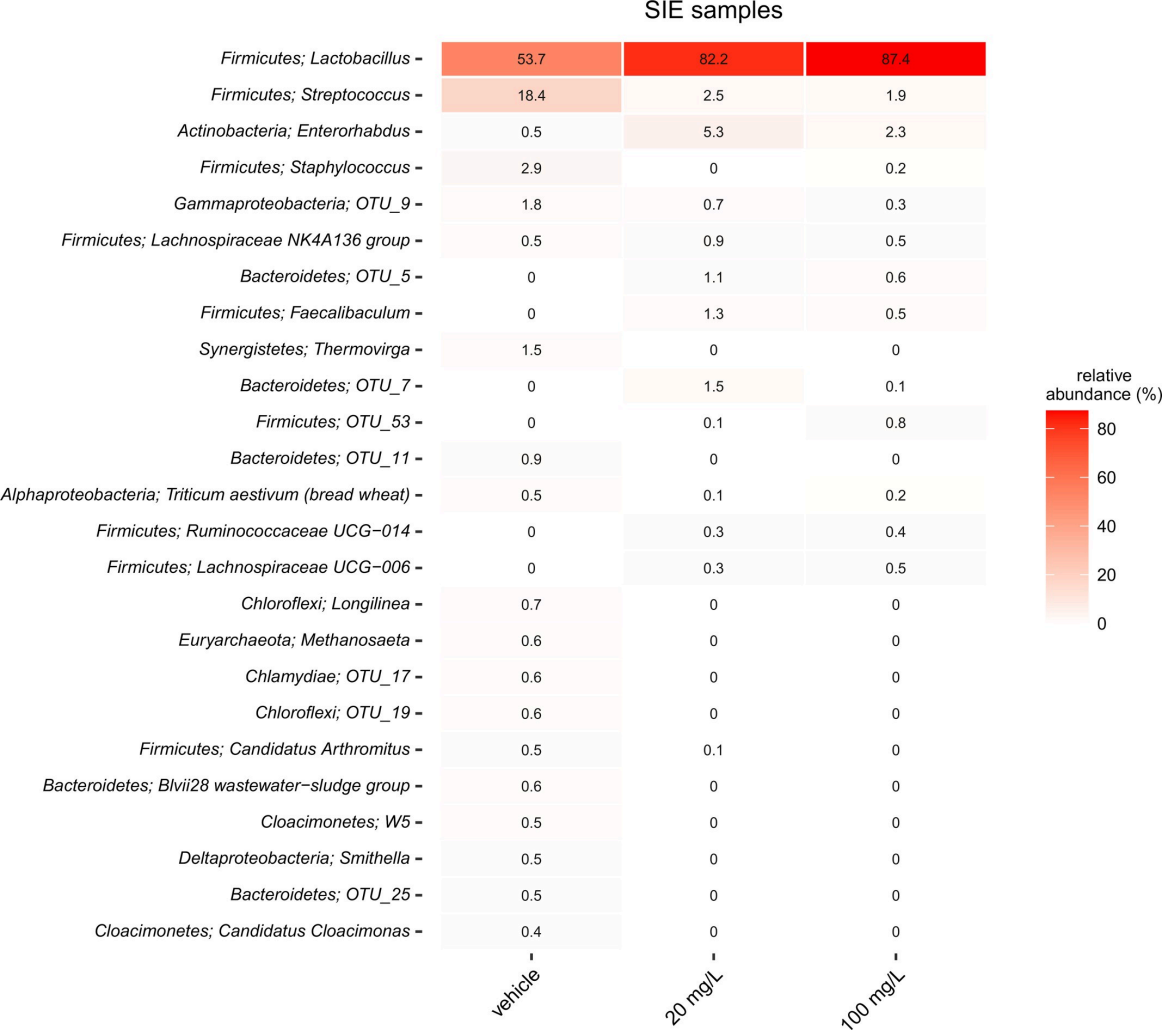
Microbial composition in small intestine epithelial (SIE) samples. The figure shows a heatmap of the 25 most abundant genera in small intestinal epithelial samples. Where available the OTU’s phylum classification is provided along with genus, and if no genus level classification could be obtained, the lowest assigned taxonomic classification is given. Values are shown as normalized fraction of total sequences (%).

As 100 mg/kg bw of JBC 1847 was well tolerated this was set as the maximum dose in the four-day treatment experiment. After four days of treatment, the microbiota species distribution seemed to differ only between the treatment group for the species of *Lactobacillus* and *Streptococcus*, and this change was more pronounced in the intestinal samples as compared to the faecal samples. However, the difference was not statistically significant, most likely due to the limited number of mice per group. (Figs 4 and 5).

The average CFU/g fecal sample before treatment was determined to be 9.6 × 10^9^, 6.2 × 10^9^ and 6.6 × 10^9^ for the vehicle group, 20 mg/kg and 100 mg/kg bw JBC 1847-treatment groups, respectively. There were no significant changes in the corresponding numbers after treatment (4.8 × 10^9^, 4.4 ×10^9^ and 6.8 × 10^9^, respectively, p = 0.51).

## Discussion

There is an urgent need for novel antimicrobials to ensure that we will be able to treat bacterial infection in the future [22]. JBC 1847 is a novel, synthetic compound that previously has been proven highly effective in decreasing the bacterial load of MRSA in an *in vivo* wound model [10]. As JBC 1847 does not pass the BBB, it was speculated whether it also have limited passage over the epithelial lining of the gastro-intestinal (GI) tract, and hence could be used to target pathogens within the GI, such as *C*. *difficile*. The *in silico* results supported that there would be only a limited systemic availability of the compound after oral administration (Table 1). The MTD also confirmed that a very high repeated dose of JBC 1847 could be tolerated without any clinical manifestations of discomfort in mice. This indicates that the plasma-concentration after oral administration is limited to a concentration less than the concentrations associated with clinical discomfort, which is supported by the *in silico* prediction of a maximal plasma-concentration of 0.046. In the *in vitro* cytotoxicity assay concentrations up to seven times MIC did not affect the TEER negatively, indicating a high tolerance of Caco-2 cells, as a mean for intestinal epithelial cells, to the JBC 1847. The tolerance was even higher when considering MTS/PMS assay in which only cells exposed to a concentration of 58.1 μg/mL (100 μM) (12 times MIC) of JBC 1847 showed a statistically significant reduction in cell metabolic activity (Fig 2). The MTS/PMS response is an overall surrogate measure of cell viability (metabolic activity), while cell monolayer barrier properties (measured as electrical resistance) and the LDH release reflects earlier stress responses. This may explain the discrepancy between the MTS/PMS assay compared to the TEER measurements and the LDH activity assay (Fig 3) in the present study.

No changes of the consistency of the fecal material was during the three days exposure trial, indicating that the compound do not cause instant dysbiosis. The clinical findings of non-dysbiosis was supported by the microbiota analysis. There were only few changes in the microbiota composition of fecal samples/small intestinal samples between JBC 1847 treated and placebo treated mice after four days of daily treatment, and none of these changes were statistically significant. There was, however, a tendency to a JBC 1847 concentration-depended increase of *Lactobacillus* spp, especially pronounced in the small intestine (Fig 5). As the data did not indicate an absolute lower number of bacteria, the increase of the relative abundance of *Lactobacillus* spp. is assumed to constitute an absolute increase, in exchange for a lower abundance of *Streptococcus* spp. (Fig 5). In general, *Lactobacillus* spp. are recognized as gut-health promoting organisms [23] and a number of commercially available pre- and probiotics aim to increase the abundance of *Lactobacillus* in the gut [24]. This is also the case for some species of *Streptococcus*, e.g. *S*. *thermophiles* [25] whereas other types of Streptococci have been frequently associated with disease [26]. For *S*. *halichori*, now considered an emerging pathogen, the intestine has been suggested as an important niche [27]. Taken together, these results does not give rise to concern regarding dysbiosis-inducing properties of JBC 1847 and it *may* even give rise to a more health-promoting microbiota with the increased level of *Lactobacillus*. The clinical importance of this observation must, however, be further investigated before scientific conclusions can be drawn.

In conclusion, this initial screening of JBC 1847 as an oral antimicrobial shows promising results against *Clostridium difficile*. Future studies on the efficacy in JBC 1847 experimental intestinal models of CDI are encouraged.
